# Species limits and phylogeography of *Newportia* (Scolopendromorpha) and implications for widespread morphospecies

**DOI:** 10.3897/zookeys.510.8573

**Published:** 2015-06-30

**Authors:** Gregory D. Edgecombe, Varpu Vahtera, Gonzalo Giribet, Pipsa Kaunisto

**Affiliations:** 1 Department of Earth Sciences, The Natural History Museum, Cromwell Road, London SW7 5BD, UK; 2 Zoological Museum, Department of Biology, University of Turku, Turku FI-20014, Finland; 3 Museum of Comparative Zoology, Department of Organismic and Evolutionary Biology, Harvard University, 26 Oxford Street, Cambridge, MA 02138, USA

**Keywords:** Scolopocryptopidae, Newportiinae, Neotropics, phylogeny

## Abstract

The genus *Newportia* Gervais, 1847, includes some 60 nominal species distributed in the Caribbean islands and from Mexico to central South America. Modern keys to species and subspecies are available, greatly facilitating identification, but some species are based on few specimens and have incomplete documentation of taxonomically-informative characters. In order to explore genetic variability and evolutionary relationships within geographically-widespread morphospecies, specimens of Newportia (Newportia) stolli (Pocock, 1896) and Newportia (Newportia) divergens Chamberlin, 1922, two nominal species distinguished principally by differences in suture patterns on T1, were sequenced for mitochondrial 16S rRNA and cytochrome *c* oxidase subunit I (COI) genes from populations in southern Mexico, Guatemala, Honduras and Brazil. Newportia (Newportia) stolli is paraphyletic with respect to Newportia (Newportia) divergens within a clade from Guatemala, Honduras, and Chiapas (Mexico), most trees being consistent with a single loss of a connection between the anterior transverse suture on T1, whereas specimens of “Newportia (Newportia) stolli” from Brazil are not closely allied to those from the Mesomerican type area. The widespread morphospecies Newportia (Newportia) monticola Pocock, 1890, was sequenced for the same loci from populations in Costa Rica, Colombia and Brazil, finding that specimens from these areas do not unite as a monophyletic group. Samples of Newportia (Newportia) oreina Chamberlin, 1915, from different regions of Mexico form geographic clusters that resolve as each other’s closest relatives. These results suggest that some widespread species of *Newportia* may be taxa of convenience more so than natural groupings. In several cases geographic proximity fits the phylogeny better than taxonomy, suggesting that non-monophyletic species do not result from use of inappropriate molecular markers. Molecular identification is possible for specimens missing taxonomically informative morphological characters, notably damaged specimens that lack the ultimate leg pair, a protocol that may also apply to other taxonomically difficult genera that are prone to damage (such as *Cryptops*).

## Introduction

*Newportia* Gervais, 1847 is a species-rich Neotropical genus that belongs to the family Scolopocryptopidae, encompassing blind Scolopendromorpha with 23 leg-bearing segments, pectinate second maxillary claws, and kinked and pineapple-shaped processes in the gizzard (Shelley and Mercurio 2005; Koch et al. 2009, 2010). *Newportia* has until recently been classified as one of two genera in the subfamily Newportiinae, distinguished from *Tidops* Chamberlin, 1915, by different forcipular structures (Chagas-Júnior 2011). Phylogenetic analyses based on multi-locus molecular sequence data have, however, indicated that *Tidops* nests within *Newportia* rather than being the sister group, as does another clade that had been assigned to a separate subfamily, the Mesoamerican Ectonocryptopinae (Vahtera et al. 2013).

The geographic distribution of *Newportia* (including *Tidops*, *Ectonocryptops* Crabill, 1977, and *Ectonocryptoides* Shelley & Mercurio, 2005 as subgenera: Vahtera et al. 2013) extends from northern Mexico throughout Central America and the Caribbean islands to Paraguay. Most species of *Newportia* have tarsus 2 of the ultimate leg divided into five to nearly 40 tarsomeres, or with indistinct separation of tarsi 1 and 2. Currently some 60 nominal species or subspecies are recognised (Minelli et al. 2006 and onwards; Schileyko 2013). In many species, diagnostic features involve the spinose processes on the ultimate prefemora and femora and the number of tarsomeres, all inconvenient characters because individuals frequently lose these legs when collected.

We propose a solution to the taxonomic impediment of missing ultimate legs by using mitochondrial sequence data to supplement identifications. We also explore phylogeographic patterns within and between select species of *Newportia* from Mexico and Central America using parsimony and maximum likelihood methods. The resultant phylogenies allow the taxonomic value of purportedly diagnostic morphological characters to be evaluated and for the limits of morphospecies to be tested.

## Methods

Thirty-four specimens of *Newportia* from Mexico, Guatemala, Honduras, and Costa Rica were sorted mostly from collections made by the LLAMA (Leaf Litter Survey of Mesoamerica) project, deposited in the Museum of Comparative Zoology (MCZ), Harvard University, Cambridge Massachusetts, USA and accessible through the dedicated data base MCZbase (http://mczbase.mcz.harvard.edu). All tissues were fixed in absolute ethanol and thus were amendable to DNA sequencing.

Identifications were made using the most recent key for Newportia (Newportia) (Schileyko, 2013), supplemented with taxonomic descriptions in modern literature (Schileyko and Minelli 1998; Chagas-Júnior and Shelley 2003), standard monographs (Attems 1930), original descriptions, and examination of type material designated by R. I. Pocock in The Natural History Museum (London) and or by R. V. Chamberlin in the MCZ.

LLAMA specimens keyed to either Newportia (Newportia) monticola Pocock, 1890, Newportia (Newportia) stolli (Pocock, 1896), Newportia (Newportia) oreina Chamberlin, 1915, or Newportia (Newportia) divergens Chamberlin, 1922. All LLAMA specimens were sequenced for two mitochondrial loci: 16S rRNA and cytochrome *c* oxidase subunit I (COI). These loci were selected because they vary both within and between species, and even between individuals from geographically close populations. The 34 LLAMA samples were supplemented with Newportia (Newportia) and Newportia (Ectonocryptoides) sequences from our previous work (Vahtera et al. 2013), nine new *Newportia* specimens from five localities in Amazonas and Roraima, Brazil, and novel sequences for an individual of Newportia (Newportia) pusilla Pocock, 1893, from Ecuador (see Table 1 for morphospecies determinations and locality data).

**Table 1.** Specimens sequenced in this study and their GenBank accession numbers. Institutional abbreviation: MCZ, Museum of Comparative Zoology, Harvard University. Bold font indicates new sequence data.

Species	Voucher ID number	Lab code	Voucher	Country (State)	16S	COI	Lat. (degrees)	Long. (degrees)
*Newportia adisi*	130770	-	MCZ	Brazil (Amazonas)	KF676465	KF676506	2.93355S	59.96611W
Newportia (Ectonocryptoides) quadrimeropus	130826	-	MCZ	Mexico (Jalisco)	HQ402494	HQ402546	-	-
*Newportia collaris*	18827	95a	MCZ	Brazil (Roraima)	**KP099547**	**KP099504**	0.99185N	62.15915W
*Newportia divergens*	98078	72	MCZ	Honduras	**KP099524**	**KP099481**	14.45748N	89.06819W
*Newportia divergens*	99129	75	MCZ	Honduras	**KP099525**	**KP099482**	14.45603N	89.06904W
*Newportia divergens*	98978	81	MCZ	Honduras	**KP099526**	**KP099483**	15.69449N	86.86339W
*Newportia divergens*	99154	82	MCZ	Honduras	**KP099527**	**KP099484**	14.48139N	87.53225W
*Newportia divergens*	88191	85	MCZ	Guatemala	**KP099528**	**KP099485**	14.5357724N	90.69427782W
*Newportia divergens*	89343	101	MCZ	Guatemala	**KP099529**	**KP099486**	14.94704N	89.27627W
*Newportia divergens*	89474	105	MCZ	Guatemala	**KP099530**	**KP099487**	14.53256659N	90.15252622W
*Newportia ernsti ernsti*	18828	94	MCZ	Brazil (Roraima)	**KP099522**	**KP099479**	1.01113S	62.11409W
*Newportia ernsti ernsti*	105917	-	MCZ	Dominican Republic	JX422692	JX422669	-	-
*Newportia longitarsis stechowi*	130774	LP2871	AMNH	French Guiana	JX422693	JX422670	4.506277N	52.058305W
*Newportia monticola*	130778	-	MCZ	Colombia	JX422694	JX422671	5.7095080242N	73.4601469617W
*Newportia monticola*	80065	40	MCZ	Costa Rica	**KP099531**	**KP099488**	8.40667N	83.32833W
*Newportia monticola*	80743	49	MCZ	Costa Rica	**KP099532**	**KP099489**	8.78658N	82.95987W
*Newportia monticola*	81355	55	MCZ	Costa Rica	**KP099533**	**KP099490**	8.94997N	82.83375W
*Newportia monticola*	21666	91	MCZ	Brazil (Roraima)	**KP099534**	**KP099491**	1.01113S	62.11409W
*Newportia oreina*	94265	57	MCZ	Mexico (Tamaulipas)	**KP099535**	**KP099492**	23.0344N	99.18697W
*Newportia oreina*	94726	58	MCZ	Mexico (Oaxaca)	**KP099536**	**KP099493**	17.89844N	96.36253W
*Newportia oreina*	94185	59	MCZ	Mexico (Tamaulipas)	**KP099537**	**KP099494**	23.0233N	99.2883W
*Newportia oreina*	93765	60	MCZ	Mexico (Tamaulipas)	**KP099538**	**KP099495**	23.0611N	99.21564W
*Newportia oreina*	93666	62	MCZ	Mexico (Tamaulipas)	**KP099539**	**KP099496**	23.00835N	99.28511W
*Newportia oreina*	95181	66	MCZ	Mexico (Oaxaca)	**KP099541**	**KP099500**	17.65934N	96.33426W
*Newportia oreina*	93981	68	MCZ	Mexico (Oaxaca)	**KP099540**	**KP099497**	17.89844N	96.36253W
*Newportia pusilla*	18758	86	MCZ	Ecuador	**KP099542**	**KP099498**	0.6083333S	77.8825W
*Newportia pusilla*	18824	90	MCZ	Brazil (Amazonas)	**KP099543**	**KP099499**	2.93349S	59.96895W
*Newportia* sp.	81282	54	MCZ	Costa Rica	**KP099544**	**KP099501**	8.94997N	82.83375W
*Newportia* sp.	18822	89b	MCZ	Brazil (Roraima)	**KP099545**	**KP099502**	0.99539S	62.15904W
*Newportia* sp.	18825	92	MCZ	Brazil (Roraima)	**KP099546**	**KP099503**	1.02897S	62.08722W
*Newportia stolli*	106516	37	MCZ	Guatemala	**KP099510**	**KP099467**	14.91852N	91.10458W
*Newportia stolli*	81361	42	MCZ	Guatemala	**KP099511**	**KP099468**	15.1144N	89.68046667W
*Newportia stolli*	81360	44	MCZ	Mexico (Chiapas)	**KP099505**	**KP099462**	16.13853333N	90.90146667W
*Newportia stolli*	79982	47	MCZ	Mexico (Chiapas)	**KP099506**	**KP099463**	16.96385N	91.59313W
*Newportia stolli*	80143	48	MCZ	Guatemala	**KP099512**	**KP099469**	15.0583333N	89.676667W
*Newportia stolli*	80175	50	MCZ	Mexico (Chiapas)	**KP099507**	**KP099464**	17.17536N	93.14939W
*Newportia stolli*	81363	52	MCZ	Mexico (Chiapas)	**KP099508**	**KP099465**	16.97416667N	91.58591667W
*Newportia stolli*	80208	53	MCZ	Mexico (Chiapas)	**KP099509**	**KP099466**	16.75181N	92.68267W
*Newportia stolli*	99225	71	MCZ	Guatemala	**KP099514**	**KP099471**	15.08405N	89.94991W
*Newportia stolli*	99279	78	MCZ	Guatemala	**KP099513**	**KP099472**	15.07708N	89.94795W
*Newportia stolli*	18826	88a	MCZ	Brazil (Roraima)	**KP099520**	**KP099477**	1.02897S	62.08722W
*Newportia stolli*	18830	93a	MCZ	Brazil (Roraima)	**KP099521**	**KP099478**	1.01113S	62.11409W
*Newportia stolli*	18827	95b	MCZ	Brazil (Roraima)	**KP099523**	**KP099480**	0.99185N	62.15915W
*Newportia stolli*	89566	99	MCZ	Guatemala	**KP099515**	**KP099470**	15.21241135N	90.21480799W
*Newportia stolli*	89321	100	MCZ	Guatemala	**KP099516**	**KP099473**	16.44568931N	89.54981728W
*Newportia stolli*	89306	102	MCZ	Guatemala	**KP099517**	**KP099474**	17.24033736N	89.62094017W
*Newportia stolli*	89355	103	MCZ	Guatemala	**KP099518**	**KP099475**	15.21318939N	90.21921316W
*Newportia stolli*	89606	104	MCZ	Guatemala	**KP099519**	**KP099476**	16.44147064N	89.53447W
*Newportia stolli*	130787	-	MCZ	Guatemala	KF676467	KF676508	15.0833333N	89.9441666W
*Cryptops punicus*	130604	-	MCZ	Italy	KF676461	KF676503	40.01471N	9.22261E
*Scolopocryptops mexicanus*	105626	-	MCZ	Ecuador	JX422703	JX422679	1.336111N	77.263055W

Total DNA was extracted from the legs utilizing the NucleoSpin®Tissue kit (Macherey-Nagel). Samples were incubated overnight. PCR amplifications were performed with illustra TM PuReTaq TM Ready-To-GoTM PCR Beads (GE Healthcare). The COI fragments were amplified using primer pair HCO1490 (Folmer et al. 1994) and HCOout (Carpenter and Wheeler 1999) and the 16S rRNA fragments using primer pair 16Sa/16Sb (Xiong and Kocher 1991; Edgecombe et al. 2002). The normal amplification cycle for COI consisted of an initial denaturation step (2 min at 95 °C), followed by 35 cycles of denaturation (1 min at 95 °C), annealing (1 min at 43 °C) and extension (1.5 min at 72 °C), followed by a final extension step (4 min at 72 °C). For the 16S rRNA fragment the cycle consisted of an initial denaturation step (2 min at 94 °C), followed by 35 cycles of denaturation (30 s at 94 °C), annealing (30 s min at 43 °C) and extension (1 min at 72 °C), followed by a final extension step (7 min at 72 °C). Visualization of the PCR products was done by 1 % agarose electrophoresis using Midori Green Advanced DNA Stain and FastGene® GelPic LED Box (Nippon Genetics, GmbH).

Samples were purified using ExoSAP-IT (Affymetrix) and sent to FIMM (Institute for Molecular Medicine Finland) for sequencing. Chromatograms were visualized and assembled using Sequencer 5.0.1 (Gene Codes Corp., Ann Arbor, Michigan, USA). Sequence alignment editor Se-Al (Rambaut 1996) was used to visualize the sequences simultaneously. GenBank registrations for new sequences are listed in Table 1.

Parsimony analysis was conducted with POY ver. 5.1.1 (Wheeler et al. 2014) run in 16 nodes in the high-performance supercluster Taito at CSC (IT-Center of Science), Finland. A timed search of three hours was first performed on the unaligned data set. The resulting tree was used as the starting tree for the next round in which an additional timed search of six hours was performed. Parameter set 111 (indel/transversion and transversion/transition costs all equal) was used throughout the searches and branch lengths were reported using the newly implemented command “report (“file_name.tre”, trees:(total, branches:true))”. Nodal support was calculated using parsimony jackknifing (Farris et al. 1996).

Additional analyses used a probabilistic approach with the maximum likelihood program RAxML ver. 8.0.22 (Stamatakis 2014). For these, multiple sequence alignments (MSA) were first estimated with MUSCLE ver. 3.6 (Edgar 2004) and then trimmed using Gblocks ver. 0.91b (Castresana 2000; Talavera and Castresana 2007) to remove areas of ambiguous alignment. Since COI sequences showed no length variation, they were not trimmed in Gblocks. The amount of 16S rRNA data that remained after trimming was 59% of the original 585 positions. The two data sets were concatenated using SequenceMatrix (Vaidya et al. 2011) and the concatenated data were analyzed with RAxML in the CIPRES Science Gateway (Miller et al. 2010). A unique general time reversible (GTR) model was specified for each partition independently. Nodal support was estimated using the rapid bootstrap algorithm (applying the Majority Rule Criterion) using the GTR-CAT model (Stamatakis et al. 2008).

## Results

The combined analysis of both COI and 16S fragments using parsimony as the optimality criterion resulted in two most parsimonious (MP) trees of length 4625 steps. The strict consensus tree (Fig. 2) shows these two trees are almost identical, differing only in the placement of two Brazilian specimens of Newportia (Newportia) stolli in relation to each other. Comparing strongly supported clades, the maximum likelihood tree (ln*L* -14054.372302: Fig. 3) shows the same major geographic and taxonomic groupings as the parsimony tree. This congruence is noteworthy because the data sets analyzed under these two optimality criteria were different (unaligned in POY and analyzed using the concept of dynamic versus static homologies with some regions removed in RAxML), as are the resampling methods (jackknifing and bootstrapping, respectively). Parts of the trees that are incongruent between the two analyses involve nodes that received low resampling supports in both analyses (e.g., the positions of Newportia (Newportia) adisi and Brazilian specimen 89b relative to other species). Both analyses depict substantial branch lengths both within and between species, with only a few instances of no (or minimal) variation between specimens from the same or geographically close populations.

**Figure 1.**
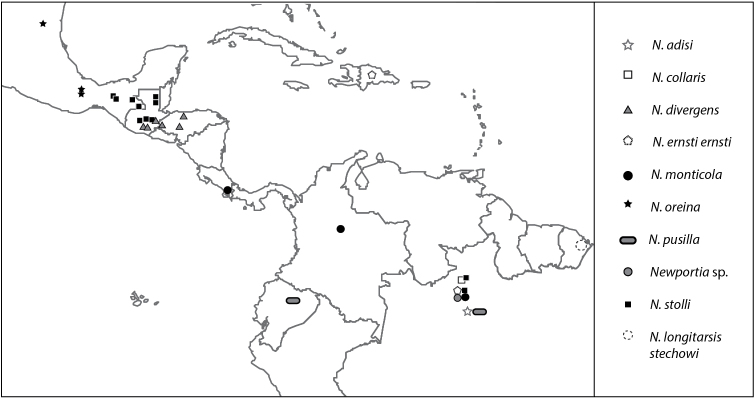
Map of Mesoamerica, the Caribbean and northern South America showing geographic distribution of *Newportia* specimens analyzed herein (see Table 1 for coordinates of samples).

**Figure 2.**
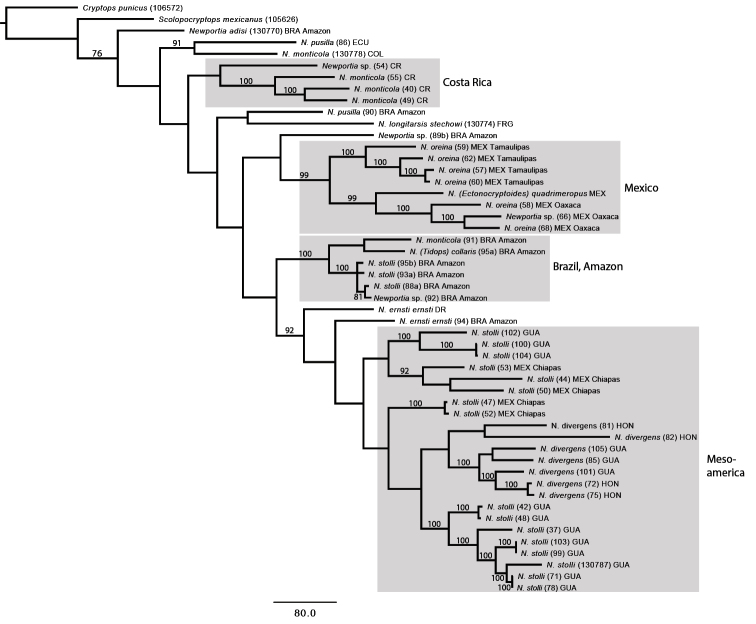
Strict consensus of two optimal cladograms for *Newportia* under parameter set 111 for parsimony (POY) analysis. Abbreviations: BRA, Brazil; COL, Colombia; CR, Costa Rica; DR, Dominican Republic; ECU, Ecuador; FRG, French Guiana; GUA, Guatemala; HON, Honduras; MEX, Mexico.

As in previous analyses based on sparser sampling for *Newportia* (Vahtera et al. 2013), *Tidops* (*Tidops collaris*) and *Ectonocryptoides* (*Ectonocryptoides quadrimeropus*) nest within *Newportia* in all analyses. Specifically, they unite with Newportia (Newportia) spp. that inhabit the same geographic region i.e., Newportia (Tidops) collaris from the Brazilian Amazon groups within a clade composed of species of Newportia (Newportia) from there, whereas Newportia (Ectonocryptoides) quadrimeropus from Jalisco, Mexico, groups with the Mexican Newportia (Newportia) oreina. These results reinforce proposals to classify *Tidops*, *Ectonocryptoides* and presumably allied *Ectonocryptops* within *Newportia* and to regard Ectonocryptopinae as subordinate to Newportiinae (Vahtera et al. 2013). The traditional classification of Newportia (Tidops) and Newportia (Ectonocryptoides) as separate genera because of their obvious phenotypic differences from *N* (*Newportia*) might have predicted that they would be markedly different from Newportia (Newportia) genetically. However, neither Newportia (Tidops) collaris nor Newportia (Ectonocryptoides) quadrimeropus depict long branch divergences from their closest relatives with respect to the studied loci, indeed being shorter than some population-level branches within species.

*Newportia oreina* consists of two geographical clades and this division is found in both parsimony and likelihood analyses; one clade consists of all specimens from Tamaulipas (JK, BS 100) and the other of ones from Oaxaca (JK 100, BS 98). Interestingly, Newportia (Ectonocryptoides) quadrimeropus forms a well-supported (JK 99, BS 73) clade with the Newportia (Newportia) oreina populations from Oaxaca, rendering Newportia (Newportia) oreina paraphyletic with respect to *Ectonocryptoides* (and presumably *Ectonocryptops*). A previous scolopendromorph phylogeny (Vahtera et al. 2013) had also indicated affinity between Newportia (Newportia) oreina and Newportia (Ectonocryptoides) quadrimeropus; analyses based on combined molecular and morphological data resolved them as sister-groups, although only one individual of each was then available. We note that *Newportia oreina* possesses a shorter tarsus than most congeners. The phylogeny interprets the ancestral condition of the ultimate leg tarsi of *Newportia* as being elongate and divided into tarsomeres, with the relatively short tarsus 2 of Newportia (Newportia) oreina being a possible precursor to the stout tarsi of the submerged taxon, “Ectonocryptopinae”. This transformation series increases the plausibility of the subclavate “ectonocryptopine” ultimate legs being derived from an ancestor with flagelliform tarsi, a result that was already strongly signaled by molecular phylogenies (Vahtera et al. 2013) and is reinforced by the current trees.

**Figure 3.**
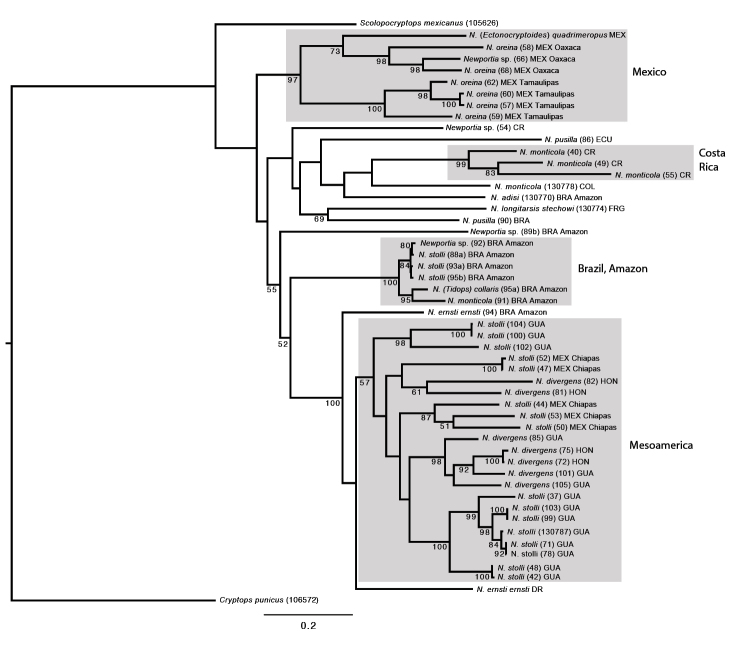
Maximum likelihood tree (ln*L* = -14054.372302). Abbreviations for countries as in Fig. 2.

A Mesoamerican clade uniting Newportia (Newportia) stolli and Newportia (Newportia) divergens from Mexico (Chiapas), Guatemala and Honduras is recovered in both parsimony and likelihood analyses (Figs 2, 3), though resampling methods did not strongly support it (JK <50, BS 57). Newportia (Newportia) divergens is resolved as monophyletic in the POY analyses but is nested within a paraphyletic Newportia (Newportia) stolli, implying a single loss of the median part of the anterior transverse suture on T1 (Fig. 2). However, there is no jackknife support for the *divergens* clade. In contrast, the likelihood analysis did not support monophyly of Newportia (Newportia) divergens; six individuals from Guatemala and Honduras resolve as a well-supported clade (BS 98), but two others from Honduras (81, 82) are grouped with two Mexican Newportia (Newportia) stolli specimens, albeit with weak nodal support.

Specimens identified as Newportia (Newportia) stolli from the Brazilian Amazon do not unite with supposed congeners from Mesoamerica but are instead most closely related to other taxa from the same region, i.e., a specimen identified as Newportia (Newportia) monticola (91) and Newportia (Tidops) collaris. This result implies that Newportia (Newportia) stolli is polyphyletic and an indistinct segmentation of ultimate tarsus 2 has multiple (convergent) origins. This character had once served as the basis for recognising a subgenus Newportia (Scolopendrides), e.g., in the classification of Bücherl (1974), but this taxon is not used in current classifications (Schileyko and Minelli 1998). We re-examined the Newportia (Newportia) stolli specimens again in light of the signal for non-monophyly in the phylogenetic analysis, attempting to recognize any morphological character(s) that would separate the specimens from Brazil from those from Mesoamerica. However, we found no distinctive characters between the samples; the specimens appear to be morphologically indistinguishable and using the existing keys they would all be identified as Newportia (Newportia) stolli with confidence.

Costa Rican specimens of Newportia (Newportia) monticola unite as a monophyletic group (JK 100, BS 99) in both analyses. In the maximum likelihood tree (Fig. 3) a Colombian specimen of Newportia (Newportia) monticola (103974) is resolved as a sister taxon to the Costa Rican clade but this relationship is not found in the parsimony tree (Fig. 2). In neither analysis did a Brazilian specimen identified as Newportia (Newportia) monticola unite with the other supposed conspecifics.

The two included specimens of Newportia (Newportia) pusilla, one from Ecuador (specimen 86) and the other from Brazilian Amazonas (specimen 90), likewise do not form a clade but instead are situated in different parts of the tree. The Brazilian specimen conforms to “Amazonian type *pusilla*” of Schileyko and Minelli (1998), characterized by rudimentary paramedian sutures on T1 (in contrast to their complete absence in other populations). Both analyses group this Brazilian specimen together with Newportia (Newportia) longitarsis stechowi but since there is no strong resampling support in either analysis (JK <50, BS 69), the question about its identity and closest relative remains unclear.

We also included a few *Newportia* specimens that could not be identified morphologically since they lacked ultimate legs, were juveniles, or did not key out to any known species. A specimen (54) from Costa Rica has a unique character combination and is apparently a distinct species but lacks its ultimate legs. In the POY analysis it groups together, although with weak support, with the Costa Rican Newportia (Newportia) monticola clade. A very distinctive Brazilian specimen (89b) with all tarsi bipartite and tarsus 2 of the ultimate leg undivided groups at the base of the Mexican Newportia (Newportia) oreina/Newportia (Ectonocryptoides) quadrimeropus clade in the parsimony analysis. However, there is poor resampling support for this grouping and it is instead allied to species with indistinctly segmented ultimate tarsus 2 and the Brazilian clade in the likelihood tree. The poor support values and topological instability under different analytical conditions render the affinities of this undescribed species uncertain.

## Discussion

Some of the specimens used in this study were either of small size because of the collection methods employed (and thus may not have been appropriate for keying using traditional criteria formulated for mature specimens) or were missing their taxonomically-informative ultimate legs. Nonetheless, several such specimens could be identified with a high degree of accuracy because their sequence data placed them within clades whose nomenclature could be established based on standard external morphological characters. An example is provided by a juvenile from Brazil (92) that is in poor condition and cannot be identified to species. However, the analysis shows it to be a juvenile of a Brazilian clade assigned to Newportia (Newportia) stolli. This approach is likely to be valuable in other groups of taxonomically-difficult centipedes that rely heavily on characters of the ultimate leg pair but often lack those legs in fixed specimens, such as *Cryptops*, where the numbers of tibial and tarsal saw teeth are fundamental taxonomic characters. The identification of developmental stages or adults without key taxonomic characters is becoming standard for many groups of animals, including other arthropod groups, such as insects (Monaghan et al. 2009; Gattolliat and Monaghan 2010) and arachnids (Fernández et al. 2014).

Some morphologically delimited species were found to be monophyletic groups, like Newportia (Newportia) divergens in the parsimony analysis, but others were paraphyletic or polyphyletic. This could be interpreted as a failure of the taxonomic characters traditionally used to delimit species or a failure in reconstructing an accurate tree by the markers selected. The second option is unlikely for the reasons outlined below, especially the biogeographical patterns exhibited in many clades where “distinct” species from the same regions tend to cluster together and not with their supposed conspecifics from other geographical regions. In particular Newportia (Newportia) stolli formed a series of geographic groupings that in part were paraphyletic with respect to sympatric species (specifically, to Newportia (Newportia) divergens in Mesoamerica) or in other cases were found to be distantly related (Brazilian “Newportia (Newportia) stolli”). The first pattern is consistent with Newportia (Newportia) stolli being a grade united by a plesiomorphy (a continuous anterior transverse suture on T1), some parts of which are most closely related to a species defined by an apomorphic state (i.e., loss of the median extent of the anterior transverse suture). The tree topology, however, suggests that the Brazilian specimens identified as Newportia (Newportia) stolli are misidentified. Newportia (Newportia) monticola is likewise a questionable taxon, the monophyletic Costa Rican group never uniting with a specimen of the same putative species from Brazil and only variably so with one from Colombia. Brazilian Newportia (Newportia) monticola and Newportia (Newportia) stolli unite in a well-supported clade (JF and BS 100), indicating that, in this instance, geography is a better predictor of relationships than taxonomy. It is noteworthy that Newportia (Newportia) stolli and Newportia (Newportia) monticola are among the most geographically widespread “species” of *Newportia*, but our results suggest that the wide distribution is partly an artifact of morphologically-based identifications. The same evidently applies to Newportia (Newportia) pusilla, a morphospecies that is regarded as ranging from St. Vincent through Colombia to the Brazilian Amazon (Schileyko and Minelli 1998; Chagas-Júnior et al. 2014). Polyphyly of this species in the molecular trees suggests that its diagnostic characters (absent or rudimentary paramedian sutures on T1 and a lack of ventral spinose processes on the ultimate leg femora) evolved convergently in different regions.

Centipede systematics, still strongly influenced by mid 20^th^ Century conceptualisations of species (see Edgecombe 2007), primarily assumes polymorphic and geographically widespread entities. The existing concepts that Newportia (Newportia) monticola and Newportia (Newportia) stolli are widespread throughout much of Central and South America exemplify where morphospecies do not appear to correspond to clades but rather are classes defined by combinations of characters. In these instances, molecular tools may prove to be invaluable for species delimitations, and novel morphological characters will need to be identified to rediagnose polyphyletic species.
